# FLINT TOOTH FAIRY (FLINT ASSESSMENT OF IN-UTERO AND AT-RISK YOUNG): THE APPLICATION OF NOVEL DENTITION ANALYSIS TO UNDERSTAND HISTORIC LEAD EXPOSURE

**DOI:** 10.51894/001c.122832

**Published:** 2024-08-30

**Authors:** Jasmin Cambri, Mallory Goldsworthy, Danielle Land, Jenny LaChance, Mona Hanna-Attisha

**Affiliations:** 1 College of Osteopathic Medicine Michigan State University

## 17

### OBJECTIVE

The Flint water crisis exposed many children to lead in the drinking water. Due to a short half-life of lead and age of recommended blood testing, surveillance blood lead screening is a poor indicator of lead in water exposure. This study aims to investigate the connection between the Flint water crisis and lead in shed teeth concentrations. Additional variables of interest include infant feeding patterns, duration of drinking water exposure, service line type, and geography.

### METHODS

After necessary IRB approvals and consents, Flint Registry enrolled children who were born in Flint from 2011-2015 and who lived within the city of Flint from April 2014-October 2015 (period on Flint River water) will be recruited. Caregivers will complete an online questionnaire about a child’s address and feeding history, including details of human milk and/or formula reconstitution during the child’s first two years of life. Shed deciduous (baby) teeth will be collected to compare risk and exposure based on child’s feeding type, line type and other geographical factors.

### RESULTS

Recruitment for study participants and deciduous teeth samples are ongoing. Baby teeth are currently being analyzed to determine weekly concentrations of calcium normalized Pb levels and other metals during pregnancy and early childhood.

### CONCLUSION

Analysis of shed teeth can be an important tool in determining historic prenatal and infant lead exposure. By understanding the relationship between teeth lead levels and infant feeding, service line type and other factors, there may be implications for safer drinking water policies and prevention practices for neurodevelopmentally vulnerable populations.

